# Integrated Genomic, Transcriptomic and Proteomic Analysis for Identifying Markers of Alzheimer’s Disease

**DOI:** 10.3390/diagnostics11122303

**Published:** 2021-12-08

**Authors:** Laura Madrid, Sandra C. Labrador, Antonio González-Pérez, María E. Sáez, The Alzheimer’s Disease Neuroimaging Initiative (ADNI)

**Affiliations:** CAEBi Bioinformática, Rio de la Plata 2, 41013 Sevilla, Spain; lmadrid@caebi.es (L.M.); sandra091999@gmail.com (S.C.L.); agonzalez@caebi.es (A.G.-P.)

**Keywords:** eQTLs, differential expression, integrative analysis, Alzheimer’s disease

## Abstract

There is an urgent need to identify biomarkers for Alzheimer’s disease (AD), but the identification of reliable blood-based biomarkers has proven to be much more difficult than initially expected. The current availability of high-throughput multi-omics data opens new possibilities in this titanic task. Candidate Single Nucleotide Polymorphisms (SNPs) from large, genome-wide association studies (GWAS), meta-analyses exploring AD (case-control design), and quantitative measures for cortical structure and general cognitive performance were selected. The Genotype-Tissue Expression (GTEx) database was used for identifying expression quantitative trait loci (eQTls) among candidate SNPs. Genes significantly regulated by candidate SNPs were investigated for differential expression in AD cases versus controls in the brain and plasma, both at the mRNA and protein level. This approach allowed us to identify candidate susceptibility factors and biomarkers of AD, facing experimental validation with more evidence than with genetics alone.

## 1. Introduction

Alzheimer’s disease (AD) is the leading cause of dementia worldwide, affecting 36 million people nowadays, and it is expected to triple its prevalence by mid-century. Familial forms of AD are caused by mutations on the amyloid-related genes PSEN1, PSEN2, and APP, while diverse candidate genes and pathways have been reported for sporadic AD, mainly provided by genome-wide association studies (GWAS) [1,2,3,4,5,6,7]. The largest risk factor for AD identified so far is the apolipoprotein E (APOE)E4 allele, conferring up to 16-fold increased risk in the homozygous state, but despite its relevance, APOE pathogenic role in AD has not been fully elucidated yet.

The clinical major hallmarks of AD are amyloid deposits and neurofibrillary tangles. Consistently with this observation, amyloid, tau protein (T-tau), and tau phosphorylated at position threonine 181 (P-tau) have been found to be present at low levels in the cerebrospinal fluid (CSF) of AD patients when compared to controls, being the only AD biomarkers currently employed in the clinical setting [8,9,10]. Considerable efforts have been put into identifying biomarkers of the disease, especially in the prodromal stage, when early intervention is expected to reduce the burden of the disease. Diverse CSF biomarkers have been proposed, including the neurofilament light protein (NFL) [11], neurogranin (Ng) [12], the neuron-specific enolase (NSE) [13], the visinin-like protein 1 (VLP-1) [14], the monocyte chemotactic protein 1 (MCP-1) [15] or the glial fibrillary acidic protein (GFAP) [16].

The identification of blood-based biomarkers is of special interest since plasma is much more accessible than CSF, and they can be applied to the screening of large populations. The fundamentals of blood-based biomarkers relate to the disruption of the blood–brain barrier, which allows the drainage of small molecules or molecules with specific transporters [17]. However, since these molecules become considerably diluted in plasma, a biochemically complex medium, well-established blood biomarkers for AD are still not in place. In fact, Amyloid β38, β40, and β42, as well as tau proteins, have also been evaluated with uneven results [18,19,20,21]. Hence, solid conclusions about the usefulness of plasma biomarkers are still a subject of active research.

Most efforts in identifying AD biomarkers until now have been based on differential expression analysis between patients and control individuals. While a large proportion of the studies used transcriptomic data, confirmation of these differences at the protein level in CSF or plasma samples has not been straightforward, and the success in identifying reliable markers of the disease has been quite limited. Integration of different layers of information can help to better understand the physiological and pathological mechanisms leading to a biological condition, from gene defects to abnormal expression of encoded or related proteins. In addition, concordance between omics serves to reduce the usually large list of candidates derived from high throughput experiments. In this paper, we aimed at identifying novel plasma biomarkers for AD, starting from GWAS summary statistics and incorporating transcriptomic and proteomic data from both the brain and blood.

## 2. Materials and Methods

### 2.1. Genome-Wide Data

Candidate Single Nucleotide Polymorphisms (SNPs) were selected from published meta-analyses of GWAS on AD cases vs control subjects [2,3,4,5], intelligence and general cognitive function [22,23], and cortical structure [24,25,26], which is related to cognitive function and neurological diseases. A total of 2978 unique SNPs were selected (Supplementary Table S1).

### 2.2. Expression Quantitative Trait Loci (eQTL) Identification

The Genotype-Tissue Expression (GTEx) v7 data were used to identify genes regulated by selected candidate SNPs in 13 brain regions (Amygdala, Anterior cingulate cortex, Caudate basal ganglia, Cerebellar Hemisphere, Cerebellum, Cortex, Frontal Cortex, Hippocampus, Hypothalamus, Nucleus accumbens basal ganglia, Putamen basal ganglia, Spinal cord cervical, and Substantia nigra). Significant variant gene–SNP pairs (i.e., those showing *p*-value below the *p*-value nominal threshold, which is defined as the empirical *p*-value of the gene closest to the 0.05 FDR threshold) were selected.

### 2.3. Brain and Blood Transcriptomics

Genes whose expression was significantly altered by the presence of candidate SNPs identified in the previous step were tested for differential expression (DE) between AD cases and controls using brain transcriptomic data. The cortex gene-expression meta-analysis included Mount Sinai Brain Bank (MSBB) dataset (frontal pole, occipital visual cortex, dorsolateral prefrontal cortex, precentral gyrus, prefrontal cortex), ROSMAP (dorsolateral prefrontal cortex), and MAYO (temporal cortex) studies, and GSE15222 [27] and GSE48350 [28] (entorhinal cortex, superior frontal cortex, post-central gyrus) datasets from the GEO repository (*n* = 1503). Hippocampal profiles were obtained from GSE11882/GSE48350 datasets [28] and the MSBB study. Blood transcriptomic datasets included ADNI and AddNeuroMed studies (*n* = 734). DE analysis between cases and controls was performed using the R package limma [29] by dataset and brain region when available. For combining DE results from the different datasets, the Random Effect Model (REM) implemented in the metaDE R tool was used. Heatmap graphs were generated with the Pheatmap R package.

### 2.4. Proteomic Data Analysis

Expression profiles of candidate genes were evaluated at the proteomic level using blood proteomics data from the ADDN study (931 proteins) and cortex proteomics from four independent datasets (BANNER, BLSA, MAYO, and MSBB; 2658 proteins). Differential protein expression analyses were performed using the limma package, with PMI, age, and sex as covariates. Meta-analysis of brain datasets was performed as described for transcriptomic data.

### 2.5. Enrichment Analysis

Enrichment analysis was performed using gPROFILER [30] on significant genes/proteins (showing REM *p*-value < 0.05 in the differential expression meta-analyses) and GTEx results. The databases being interrogated included GO, KEGG, WikiPathways, and Reactome. Path graphs were generated using the R package rrvgo.

## 3. Results

**Figure 1:**
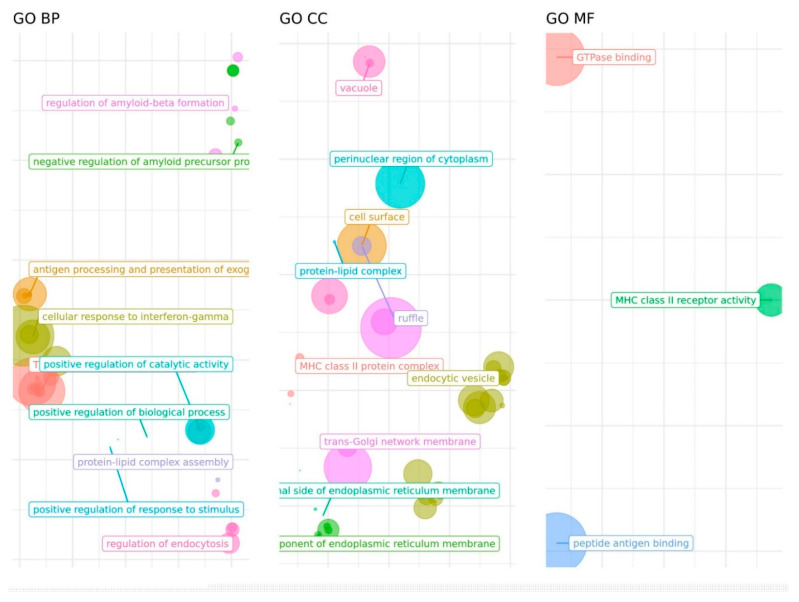
Gene Ontology (GO) categories over-represented among genes regulated by AD eQTLs. BP: Biological Process; CC: Cellular Component, MF: Molecular Function. Distances between points represent the similarity between terms. Axes are the first 2 components of applying a Principal Component Analysis (PCA) to the (di)similarity matrix. Size of the point represents the provided scores or, in its absence, the number of genes the GO term contains.

There was limited overlap between candidate SNPs from the three phenotypes: only one SNP shared by AD and cognitive performance candidate lists (rs3817334), and two SNPs in common in the cognitive performance and cortical structure lists (rs13212044 and rs13107325). Genes regulated in diverse brain tissues by the 2186th, 398th, and 412th candidate SNPs from AD, cognition, and cortical measures GWAS were explored using GTEx. We found 51, 75, and 140 regulated genes, respectively (Supplementary Tables S2–S4). Six genes from the 11p11.2 (*C1QTNF4, FAM180B, MADD, MTCH2, SLC39A13, and NDUFS3*) were found to be regulated by the AD and cognitive performance candidate SNPs, determined by the common rs3817334 polymorphism plus rs11605348 for the cognitive phenotype and 135 additional SNPs for the AD phenotype. There was no overlap between AD and cortical measures eQTL-regulated gene lists, whereas 24 shared genes were identified for the cognitive performance and cortical structure phenotypes (*ARHGAP27, ARL17A, DCC, DEPDC1B, FMNL1, GOSR1, KANSL1, KANSL1-AS1, LINC02210, LOC107984142, LOC339192, LRRC37A, LRRC37A2, MAPT, NDUFAF2, NPIPB9, PLEKHM1, RPS26, SH2B1, SPPL2C, SULT1A1, SULT1A2, SUOX,* and *TUFM*). The 51 AD genes were involved in immune-related functions, mainly driven by the HLA and *CR1 loci*, amyloid-related pathways (*ABCA7, BIN1, PICALM* genes), protein-lipid complexes (*ABCA7, APOC2, APOC4, BIN1* genes), and vesicle trafficking (*RIN3, PICALM*) (Figure 1, Supplementary Table S5). By contrast, no significant enrichment was found either for cognition or cortical genes, although the 24 shared genes were mostly related to cytoskeleton and endocytosis, neurotransmitters metabolism and oxidative phosphorylation.

**Figure 2:**
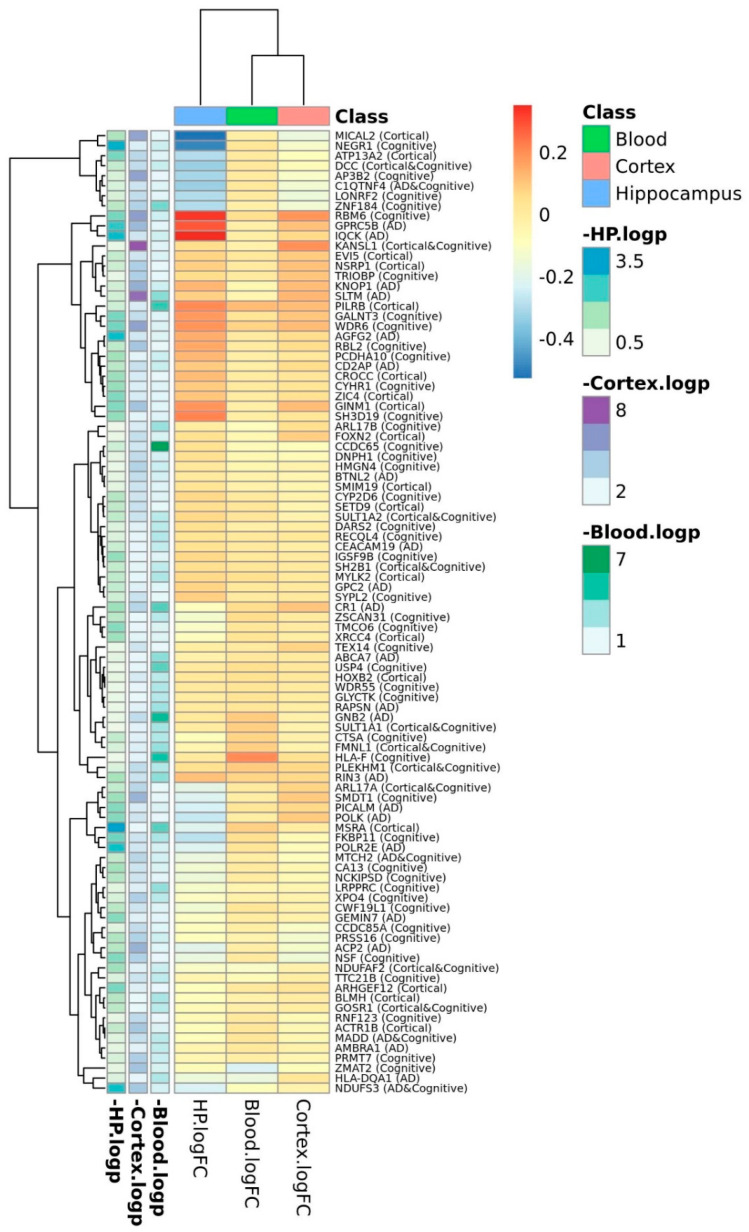
Heatmap plot of logFC expression values between AD cases and controls at the mRNA level.

The expression patterns of the genes identified by GTEx eQTL analyses were explored using blood and brain transcriptomics (Supplementary Tables S6–S8). In the blood, 10 of the 41 (24.4%) AD genes present in the expression dataset were differentially expressed in cases vs controls, while 11 of 62 (17.7%) cortical genes, and 22 of 108 (22.4%) cognitive genes (Figure 2) were also differentially expressed in blood. In the brain cortex, 16 of 48 (33.3%), 15 of 69 (21.7%), and 39 of 119 (32.8%) AD, cortical, and cognitive-available genes were found differentially expressed in the brain, respectively. Finally, in the hippocampus, 9 of 48 (18.8%), 8 of 66 (12.1%), and 14 of 119 (11.8%) AD, cortical, and cognitive genes, respectively, were differentially expressed between cases and controls.

**Figure 3:**
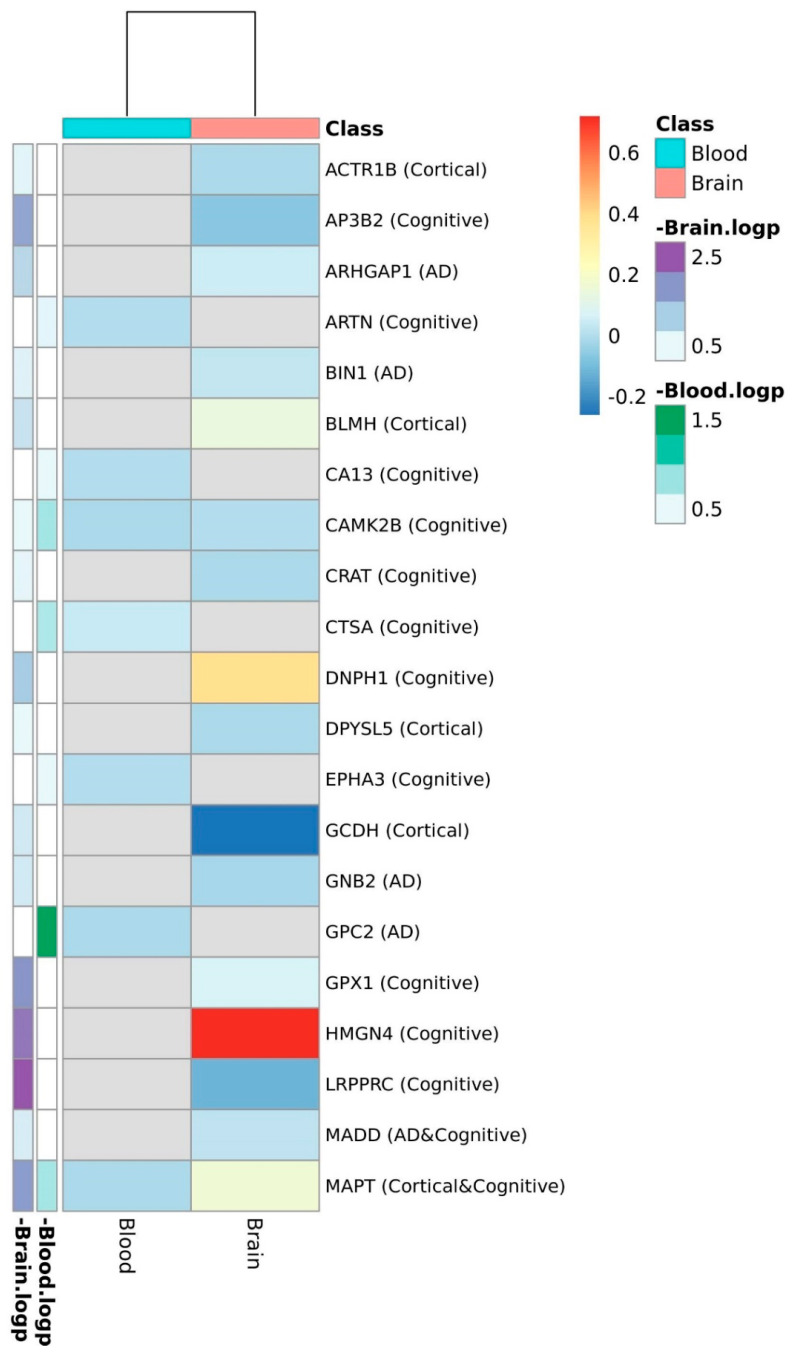
Heatmap plot of logFC expression values between AD cases and controls at the protein level. FC: Fold Change. Gene FCs were calculated as the average expression in the AD group relative to the average expression in the control group.

At the proteomic level, only 29 genes were represented in the brain and 9 in the blood from the total list of candidates (Figure 3, Supplementary Tables S9 and S10). The single AD candidate gene represented in the plasma dataset, GPC2, was shown to be downregulated in AD cases when compared to control subjects; neither tau protein (encoded by MAPT gene) nor the other cognitive and structural candidates represented in this dataset (ARTN, CA13, CAMK2B, CTSA, EPHA3, MAPT, MST1R, and ULBP) showed differences between AD cases and controls in plasma. In the brain, the NADH Dehydrogenase NDUFS3, the glutathione peroxidase enzyme encoded by GPX1, the chromatin-associated protein HMGN4, and tau were upregulated in AD cases, whereas TUFM (Mitochondrial Elongation Factor Tu), LRPPRC (Mitochondrial Leucine-Rich PPR Motif-Containing Protein), and AP3B2 (Adaptor protein complex 3) were downregulated.

## 4. Discussion

The current approach represents an integrative analysis of multi-omic data for Alzheimer’s disease patients and control subjects, aimed at identifying blood biomarkers of this prevalent degenerative disease. Starting from candidate SNPs for AD, general cognitive function, and cortical structure reported in published meta-analyses, we used the GTEx database for identifying genes regulated by these candidate gene variants. The expression of these genes at the mRNA level in the brain and blood was explored by means of differential expression analysis between AD patients and controls. Finally, gene candidates were evaluated at the proteomic level in the blood and cortex, an analysis step hampered by the low availability of blood-based proteomic datasets and the limited range of proteins represented in high throughput proteomics compared to transcriptomics. In fact, only one AD candidate gene was represented in the ADDN blood proteomic data, the Glypican-2 (GPC2) protein, significantly downregulated in AD cases.

GPC2 is a cell surface proteoglycan that bears heparan sulfate. Heparan sulfate proteoglycans (HSPGs) are components of the cell surface and extracellular matrix, and constitute central players in the development and functions of synapses [31]. In particular, it has been reported that GPC2 levels in CSF resemble the status of adult hippocampal neurogenesis, decreasing over time according to preliminary studies [32]. Furthermore, a recent meta-analysis has shown a genome-wide significant statistical association with AD of the rs12705073 polymorphism, located in the first intron of the GPC2 gene [33,34]. The GPC2 homologue GPC5 has been shown to be downregulated in both CSF and plasma of Aβ negative MCI subjects when compared to control subjects in the Biofinder study [35]; HSPGs are present in amyloid lessons, promoting Aβ peptide and tau fibrillization and providing resistance against proteolytic breakdown [36]. GPC2 has been shown to undergo a significant upregulation when exposed to Aβ in two mouse models of AD, mainly derived from the glial cell [37,38]. In particular, GPC2 is expressed in the oligodendrocyte precursors cells (OPCs), the precursors of the myelin-producing oligodendrocyte cells. According to the ROSMAP snRNAseq data and the scREAD database, GPC2 is downregulated in OPCs from AD patients when compared with control subjects. In a previous report, we identified these cells as key drivers in AD pathogenesis [39]. In fact, myelin disturbance as an etiological agent for AD is now increasingly being studied, and a recent report confirms its key role in a murine model of AD [40]. Further studies in larger cohorts are required to validate the utility of GPC2 as an AD biomarker, and to investigate if an interaction with APOE exits in vivo, since the 34-kD glycoprotein exerts its function in lipoprotein clearance via binding to specific cell surface receptors, including the LDL receptor and diverse HSPGs. Interestingly, the two classes of membrane HSPGs, transmembrane syndecans (SDCs), and glycosylphosphatidylinositol-anchored glypicans (GPCs) have been shown to interact with ApoE modulating amyloid uptake and aggregation in a haplotype-dependent manner [41,42].

Over the past decade, the advent of high throughput omic technologies has fuelled the discovery-based approach, providing access to a large number of candidate molecules, in contrast to classical approaches, mostly focused on candidate genes from known pathways of the disease, mainly related to amyloid pathology and inflammatory response in the case of AD. Despite the application of a wide variety of sophisticated approaches for the statistical analysis of large datasets, the results have been disappointing overall, and the repertory of clinical biomarkers overall has been only slightly increased, most of them oncologic biomarkers. Strategies based on differential expression analysis have been by far the most used approach, in part because of the simplicity of logFC calculations. However, the classification accuracy of identified, differentially-expressed biomarkers is, overall, not high when validated at the proteomic level. A recent meta-analysis of proteomic candidate biomarkers for AD described six markers consistently replicated in at least three independent cohorts: alpha-2-macroglobulin (α2M)_ps_, pancreatic polypeptide (PP)_ps_, apolipoprotein A-1 (ApoA-1)_ps_, afamin_p_, insulin growth factor binding protein-2 (IGFBP-2)_ps_, and fibrinogen-γ-chain_p_ [21]. Another recent meta-analysis reported that CSF T-tau, P-tau, Aβ42, NFL, and plasma T-tau were strongly associated with AD, whereas emerging CSF biomarkers such as NSE, VLP-1, HFABP, and YKL-40 were moderately associated, and plasma Aβ42 and Aβ40 were not associated with AD [19].

In conclusion, the identification of biomarkers is particularly challenging in the neurology field, AD being a good example of this complexity. Poor clinical diagnostics, long asymptomatic prodromal stages, variability in clinical features and rates of progression, besides individual genetic susceptibility, are some determinants of this situation. The combination of different levels of information at different disease stages is now possible, thanks to the availability of high throughput multi-omics technologies, opening a new era for biomarker discovery although still limited by the availability of large untargeted proteomic datasets. This report represents a preliminary study aimed at illustrating an analysis pipeline applicable to any disease with a genetic component. Further studies are therefore required to investigate the potential of GPC2 as an AD plasma biomarker, using large populations in a clinical setting.

## Data Availability

Data is available as Supplementary material.

## Supplementary Materials

The following are available online at https://www.mdpi.com/article/10.3390/diagnostics11122303/s1, Table S1: Candidate SNPs; Table S2: GTEx results for AD candidate SNPs; Table S3: .GTEx results for cognitive performance SNPs; Table S4: GTEx results for cortical structure candidate SNPs; Table S5: Enrichment analysis of genes regulated by AD candidate SNPs; Table S6: Blood transcriptomics; Table S7: Cortex transcriptomics; Table S8: Hippocampus transcriptomics; Table S9: Blood proteomics; Table S10: Brain proteomics.
